# Bisphosphonate-treatment of secondary chronic osteomyelitis of the jaw – a case report

**DOI:** 10.1093/jscr/rjad270

**Published:** 2023-05-25

**Authors:** Diana Heimes, Peer W Kämmerer

**Affiliations:** Department of Oral and Maxillofacial Surgery, University Medical Center Mainz, 55131 Mainz, Germany; Department of Oral and Maxillofacial Surgery, University Medical Center Mainz, 55131 Mainz, Germany

**Keywords:** Secondary chronic osteomyelitis, jaw, bisphosphonate, pamidronate

## Abstract

Secondary chronic osteomyelitis (SCO) is caused by bacterial infection leading to progressive jawbone destruction. Antibiotics are used as first-line treatment; surgical therapy is usually extensive and may not provide a cure. Bisphosphonates have been reported to be successful in patients with primary nonbacterial osteomyelitis, and literature indicates promising results in SCO. A 38-year-old patient presented with a progressive mandible-destruction 17 years after wisdom tooth extraction. Several treatment attempts have been unsuccessful. Seeking a second opinion, the patient was then interdisciplinary treated with 90 mg of intravenous pamidronate every four weeks, three times. The patient did not experience side effects presenting a considerable improvement in mouth opening and reporting a dissolvent of signs of pain or infection. The MRI showed edema reduction and decreased contrast uptake. Therefore, bisphosphonate treatment of secondary chronic osteomyelitis of the jaw is a safe and effective option in selected cases after unsuccessful first- and second-line therapy.

## INTRODUCTION

Osteomyelitis of the jaw is a common disease in oral and maxillofacial surgery. The Zurich classification system is based on radiological and clinical appearance and defines three main subtypes according to the etiology and pathogenesis of the disease. The classification system distinguishes between acute, primary, and secondary chronic osteomyelitis. Entering with a bacterial trigger, acute and secondary chronic osteomyelitis are separated by a time limit definition of one month after the onset of the disease. Clinical signs may be swelling, pain, suppuration, sequestration, and fistula. Potential causes are odontogenic disease, periodontal or pulpal infection, extraction wounds, and foreign bodies.

Osteomyelitis is considered an inflammatory condition of the bone extending from the marrow to the periosteum. The treatment has profoundly changed during the last century; the introduction of antibiotics led to a decreased prevalence and an improved prognosis of patients suffering from this disease [1]. Nowadays, antibiotics are still the first-line therapy for acute and secondary chronic osteomyelitis (SCO). Since prolonged treatment is often required, antimicrobial resistances frequently demand a continual change of antimicrobial agents. Furthermore, side effects are common, and even hepatic or renal damage is possible. Ablative surgery, from local decortication to continuity defects of the jaw, is considered the definitive treatment modality in several cases where systemic antibiotics are limited in success. But those interventions can be disfiguring, resulting in loss of chewing function and life quality and sometimes demanding jaw microvascular or alloplastic reconstruction [2, 3]. In refractory cases, hyperbaric oxygen therapy has been advocated; however, evidence is scarce [3].

Bisphosphonate therapy has been described as a successful treatment modality in primary, also called nonbacterial osteomyelitis of the jaw [3]. Since nonbacterial osteomyelitis is an autoinflammatory disease, first-line treatment consists of nonsteroidal anti-inflammatory drugs (NSAIDs), alone or combined with steroid therapy. Second-line treatment options are disease-modifying antirheumatic drugs, biologics, or bisphosphonates. Here, the Childhood Arthritis and Rheumatology Research Alliance (CARRA) proposes pamidronate and zoledronate [4]. Several authors reported the successful use of bisphosphonates in patients with primary nonbacterial osteomyelitis [5–8]. A recent review analyzed the existing literature regarding the evidence of bisphosphonate treatment in such patients. It concluded that NSAIDs and bisphosphonates should ideally be combined as first-line therapy [9].

There are no clinical studies on treating SCO of the jaw using bisphosphonates or analyzing the effectiveness of different preparations. In 2020, Cheng et al. published the first case report on a patient with SCO of the jaw successfully treated with alendronic acid 70 mg once weekly [2].

Here, we report the second case of extensive refractory SCO of the jaw that failed to respond to antimicrobial treatment, surgery, and hyperbaric oxygen therapy successfully managed with intravenous bisphosphonates.

## CASE REPORT

A 38-year-old male patient presented to the Department of Oral and Maxillofacial Surgery of the University Medical Center in Mainz (Germany) seeking a second opinion for treating severe and refractory osteomyelitis of the mandible. In 2005, his wisdom teeth were removed; since then, he has suffered from local recurrent infection, pain, and swelling. In 2007 local decortication was performed without any long-term effect. Over time he was treated with four different antibiotics (amoxicillin, amoxicillin and clavulanic acid, clindamycin, and moxifloxacin) and hyperbaric oxygen therapy (2007), which led to an amelioration of symptoms for only two weeks. In 2022, the resection of the mandible and reconstruction with a microvascular free fibular graft was recommended by another clinic. Seeking a second opinion, the patient presented with a progression of his symptoms under long-term antibiosis with moxifloxacin.

Co-morbidities or regular medications were absent; the patient was a non-smoker and a non-drinker.

Extra-oral examination revealed local swelling of the left mandible, erythema, and fistula. Intra-orally, the mouth opening was limited to 1 mm, no bone was exposed, and the patient showed tenderness on palpation in the left lower jaw.

**Figure 1:**
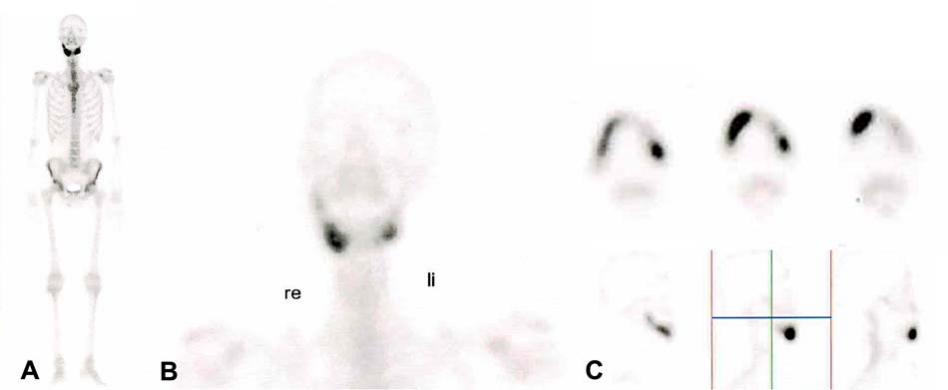
99 m Technetium scintigraphy of the patient showing an intense increased enhancement in the mandible during the late phase. This enhancement was strongest in the anterior portion of the jaw as can be seen in (B) and (C). A: Scintigraphy of the complete body. B: Scintigraphy of the head and neck in coronal direction. C: Scintigraphy of the mandible in axial sections (upper part) and in sagittal section (lower part).

**Figure 2:**
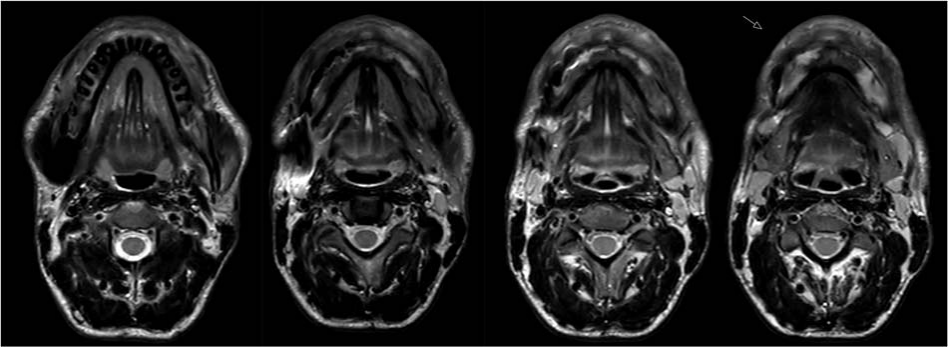
Magnetic resonance imaging (MRI) before bisphosphonate therapy (axial sections). T2 weighting of the MRI shows the high inflammatory activity especially in the anterior portion of the mandible (arrow).

**Figure 3:**
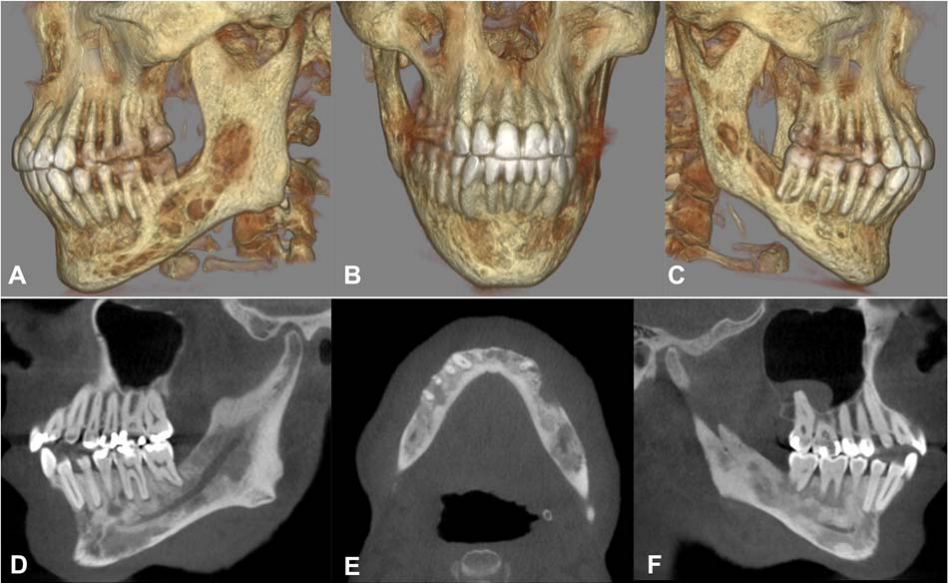
Cone beam computed tomography. A–C) Three-dimensional reconstruction showing the severe destruction of the mandible. D–F) Sagittal and axial sections showing coexisting patchy radiolucent areas within the mandible reaching up to the condyle on both sides.

In 2020, a 99 m Technetium scintigraphy was performed to rule out disseminated osteomyelitis. The scintigraphy showed no other foci of enhancement but the mandible with increased inflammatory activity in the anterior portion of the jaw (Fig. 1). Magnetic resonance imaging (MRI) was performed to analyze the localization and extent of the disease. In Fig. 2, a high inflammatory activity, resembled by a strong local enhancement in the T2 weighting, was seen, reaching from the right condyle to the left. Cone beam computed tomography (CBCT) confirmed a widespread patchy radiolucency extending to both left and right mandibular condyles (Fig. 3). Hard tissue biopsies were taken to rule out malignancy due to the disease’s extensive destruction and chronic course. Blood assays were also requested.

**Figure 4:**
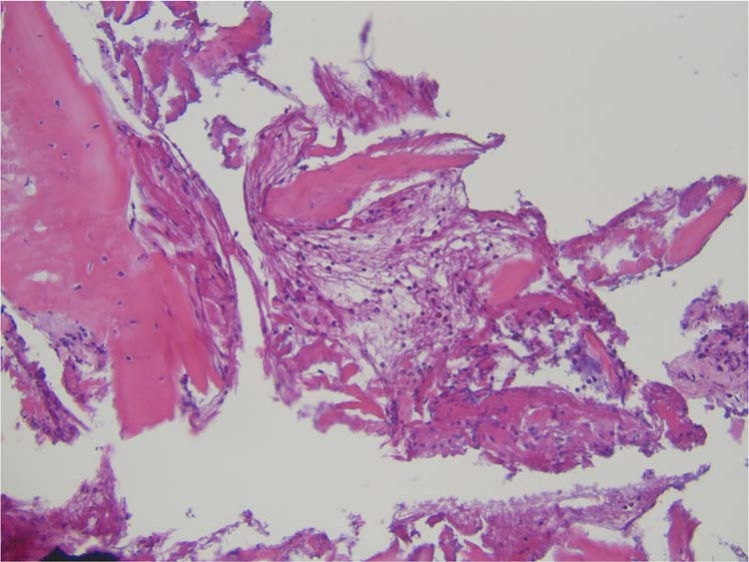
Histopathological specimen showing bone with signs of remodeling and chronic inflammation.

The histopathological analysis reported compact and cancellous bone with signs of remodeling, fibrous medulla, and a slight chronic inflammation compatible with osteomyelitis (Fig. 4). Laboratory results showed elevated liver function test results (due to the long-term antimicrobial therapy) and high inflammatory markers (c-reactive protein) but no change in the blood cell count, especially leucocytes.

Regarding the constant progression of the destructive disease being resistant to first- and second-line therapy, the lack of further options led to another clinic’s proposition of total mandibulectomy. Since this approach was deemed radical, considering the benign condition, it was agreed to trial 90 mg of intravenous pamidronate every four weeks, three times in total, after an interdisciplinary conference involving the Department of Rheumatology. This decision was based on the recommendation of the Childhood Arthritis and Rheumatology Research Alliance (CARRA) concerning the treatment of patients with primary chronic osteomyelitis. A detailed explanation was given to the patient, especially regarding the off-label use of the medication and potential side effects. After written consent was obtained, blood samples were taken to check for renal and hepatic function and cell count of the blood components and electrolytes. According to the product information, common side effects of pamidronate are anemia, thrombocytopenia, leukopenia, hypocalcemia, hypophosphatemia, atrial fibrillation, hypertonia, nausea, arthralgia, acute kidney failure, and fever.

Pamidronate was administered in 250 ml sodium chloride (0.9%) over two hours using an infusion pump with a 2 ml/min rate. The patient was asked to stay hydrated with at least 1 liter of water each before and after the infusion. Blood samples were taken and analyzed regarding the abovementioned parameters one day after the administration. No significant changes in the laboratory results were found, nor have the patient show any signs of side effects mentioned in the product information. Beginning five days after the first treatment, the patient reported significantly less pain, reduced swelling, less suppuration, and improved maximum mouth opening. Four weeks after the first treatment, he presented with a maximum mouth opening of 2.3 cm, no signs of infection, and no tenderness (Fig. 6). The laboratory inflammatory parameters (c-reactive protein – CRP) dropped from 65 mg/dl before to 5.5 mg/dl after the first treatment; during the further course of the therapy, CRP even normalized to 1.5 mg/dl.

**Figure 5:**
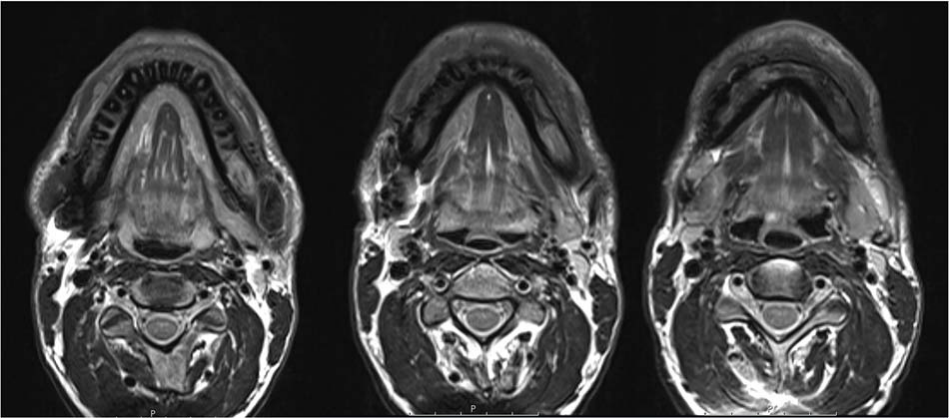
Magnetic resonance imaging (MRI) 3 months after bisphosphonate therapy. T2 weighting shows the significant reduction of inflammatory activity within the mandible after bisphosphonate treatment.

The MRI performed three months after the last pamidronate infusion revealed a significant reduction of enhancement in the mandible with some patchy hypointense areas remaining (Fig. 5).

**Figure 6:**
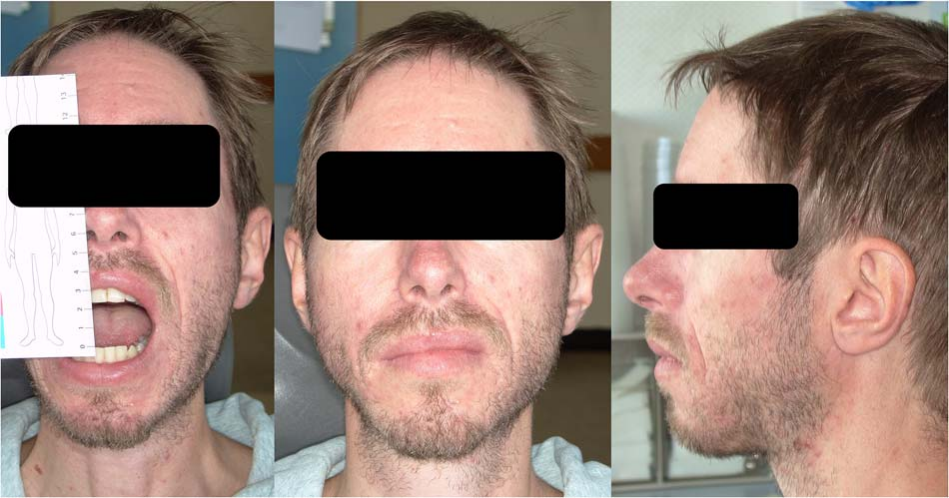
Clinical appearance with a maximum mouth opening of 2.5 cm after three cycles of pamidronate therapy.

## DISCUSSION

Osteomyelitis is an inflammatory condition of the bone in the facial skeleton, usually present in the tooth-bearing parts. Local factors such as trauma, tooth extraction, and dental infection have been identified as risk factors since the tooth creates direct contact between the oral cavity and the bone. Individual predisposing factors are deemed to be diabetes, anemia, malnutrition, and other diseases compromising the immune system [10]. Chronic osteomyelitis of the mandible was first described by Pell et al. [11] in 1955. It is defined as a multifactorial disease with complex etiology and a clinical course presenting with chronic pain and inflammation with periods of exacerbation [10]. Severe complications, such as nerve damage and pathological fractures, have been reported. The management is often complicated, with antibiotics and NSAIDs being used to control symptoms; however, they seldom reduce the frequency of recurrences and the duration of the disease. The length of antimicrobial therapy is controversial. Baur et al. recommended determining the treatment time according to the patient’s symptoms and laboratory inflammation markers [10]; others suggested 4 or 8 weeks of antibiotic treatment after surgery [12, 13]. Several authors have proposed surgical management as an integral part of the therapy since antibiotic treatment is thought only palliative when used alone [9, 13]. Baur et al. reported 24 cases in which radical surgical resection in combination with antibiotic therapy resulted in complete healing of the SCO. Only three patients remained symptomatic after the treatment [10]. Radiology typically indicates sequestra formation, irregular bone lesions, and proliferative periostitis; however, it takes some time for the bone to demineralize before such signs become detectable. Due to the refractory nature of the disease, inadequate surgical removal may complicate further treatment, prolong the treatment period, and increase the cost. Eventually, subtherapeutic conservative management of the necrotic bone could lead to morbidity comparable to primary aggressive surgical management. Since the surgical resection can be disfiguring, resulting in loss of chewing function and life quality, and sometimes demanding jaw microvascular or alloplastic reconstruction, adjuvant therapies would be favorable. Hyperbaric oxygen therapy has been proposed as an alternative treatment option in therapy-resistant cases. Still, controversies in international literature and the lack of high-quality clinical studies question its use [2, 3].

Chronic non-bacterial osteomyelitis is a rare auto-inflammatory bone disorder primarily affecting children. Clinical signs are heterogeneous, ranging from asymptomatic bone lesions to chronic recurrent multifocal osteomyelitis. While chronic non-bacterial osteomyelitis can be present in all bones, the jaw is rarely affected, primarily occurring in adolescent patients [9]. To date, there are no guidelines regarding the treatment of this disease. NSAIDs are considered the first-line therapy showing better results in children than in adults [14]. Aside from oral steroids and biologics, bisphosphonates are considered the second-line therapy in NSAID-refractory cases and have been used for decades. Today, intravenous zoledronic acid is the most commonly used bisphosphonate [15, 16]. Improved symptoms, as evaluated by the quality of life and pain relief, are typically seen after the first infusion lasting 1 to 4 years [14, 15]. In its 2018 proceedings, the Childhood Arthritis and Rheumatology Research Alliance (CARRA) recommends three different treatment regimens for patients unresponsible to NSAIDs. One of those regimens mentioned is the treatment with bisphosphonates like pamidronate [17]. Their mechanism in osteomyelitis is still unclear but is thought to be related to decreased osteoblast and osteocyte apoptosis, reduced bone resorption, and increased bone mineralization [17, 18]. Bisphosphonates have been used since the 1990s to treat osteoporosis and bone metastases, hypercalcemia, and Paget disease [19]. Like native pyrophosphate, bisphosphonates inhibit bone resorption by attaching to hydroxyapatite binding sites on the bone, particularly in areas with active resorption. Pamidronate inhibits farnesyl pyrophosphate synthase; this enzyme acts to promote attachment of the osteoclast to the bone, which, as a result, detaches the osteoclast from the bone surface. It is typically administered as 30 to 60 mg by slow intravenous infusion every 3 to 6 months to treat hypercalcemia of malignancy, Paget disease, and bone metastasis [19].

The ischemic nature of chronic osteomyelitis produces an area with less oxygen saturation resulting in a predominantly anaerobic bacterial environment. The low blood diffusion rates within the avascular bone further hinder the penetration of antimicrobial agents. While high serum rates indicate effective doses, local concentration within the affected bone may be insufficient. Furthermore, it has been hypothesized that a local disturbance between osteoblastic and osteoclastic activity may underly the disease process [2]. The mechanism of bacterial infection leading to SCO has not yet been identified. However, it is suggested that bacterial products can directly activate osteoclast activity. The positive effects of bisphosphonates in patients with primary chronic osteomyelitis strengthen the argument of osteoclasts being pivotal in the pathogenesis of osteomyelitis, which led to the assumption, that it might also be helpful in SCO [2]. The favorable results in this case demonstrate the potential application without surgical intervention. Since medication-related osteonecrosis of the bone is caused by bisphosphonates, their use in osteomyelitis remains controversial. Even some authors have suggested a higher risk of developing osteonecrosis after bisphosphonate therapy in patients with osteomyelitis [2, 20, 21].

The current case demonstrates the successful management of secondary chronic osteomyelitis using intravenous high-dose pamidronate over a short period. Since this is the second case reported in international literature, firm recommendations can only be proposed. The treatment with bisphosphonate might be a new approach in refractory cases and needs to be further investigated in clinical studies.

## Data Availability

The data are available from the corresponding author on reasonable request.
